# PER2-mediated ameloblast differentiation via PPARγ/AKT1/β-catenin axis

**DOI:** 10.1038/s41368-021-00123-7

**Published:** 2021-05-19

**Authors:** Wushuang Huang, Xueqing Zheng, Mei Yang, Ruiqi Li, Yaling Song

**Affiliations:** The State Key Laboratory Breeding Base of Basic Science of Stomatology (Hubei-MOST) and Key Laboratory of Oral Biomedicine Ministry of Education, School and Hospital of Stomatology, Wuhan University, Wuhan, China

**Keywords:** Developmental biology, Cell biology

## Abstract

Circadian rhythm is involved in the development and diseases of many tissues. However, as an essential environmental regulating factor, its effect on amelogenesis has not been fully elucidated. The present study aims to investigate the correlation between circadian rhythm and ameloblast differentiation and to explore the mechanism by which circadian genes regulate ameloblast differentiation. Circadian disruption models were constructed in mice for in vivo experiments. An ameloblast-lineage cell (ALC) line was used for in vitro studies. As essential molecules of the circadian system, *Bmal1* and *Per2* exhibited circadian expression in ALCs. Circadian disruption mice showed reduced amelogenin (AMELX) expression and enamel matrix secretion and downregulated expression of BMAL1, PER2, PPARγ, phosphorylated AKT1 and β-catenin, cytokeratin-14 and F-actin in ameloblasts. According to previous findings and our study, BMAL1 positively regulated PER2. Therefore, the present study focused on PER2-mediated ameloblast differentiation and enamel formation. *Per2* knockdown decreased the expression of AMELX, PPARγ, phosphorylated AKT1 and β-catenin, promoted nuclear β-catenin accumulation, inhibited mineralization and altered the subcellular localization of E-cadherin in ALCs. Overexpression of PPARγ partially reversed the above results in *Per2*-knockdown ALCs. Furthermore, in in vivo experiments, the length of incisor eruption was significantly decreased in the circadian disturbance group compared to that in the control group, which was rescued by using a PPARγ agonist in circadian disturbance mice. In conclusion, through regulation of the PPARγ/AKT1/β-catenin signalling axis, PER2 played roles in amelogenin expression, cell junctions and arrangement, enamel matrix secretion and mineralization during ameloblast differentiation, which exert effects on enamel formation.

## Introduction

Circadian rhythm is involved in most physiological processes.^1^ A circadian clock in the suprachiasmatic nucleus responding to light can be entrained by light/dark (LD) cycles, which acts as a master clock to synchronize peripheral clocks that reside in peripheral tissues such as liver, kidney, heart and blood vessels.^2,3^ The peripheral clocks are also independently regulated by physiological stimuli such as feeding.^1^ The encoded proteins of the core clock genes CLOCK and BMAL1 heterodimerize to activate the transcription of clock-controlled genes, which include *Period* (*PER1, PER2, PER3*) and *Cryptochrome* (*CRY1, CRY2*) genes.^4,5^ Upon accumulation to a critical level, PER and CRY proteins translocate into the nucleus to repress the transcriptional activity of *CLOCK* and *BMAL1*, thereby inhibiting their own transcription.^4,6^ Disturbance in the circadian rhythm resulting in dysregulation of circadian genes causes metabolic disorders and mental illness, such as obesity and diabetes, and increases the risk of cancer and Alzheimer’s disease.^7,8^

Circadian rhythm plays roles in physiological and behavioural processes in organisms. However, the association between circadian rhythm and tooth development, especially enamel development, has not been fully elucidated. As the hardest tissue in the human body, dental enamel forms to protect the tooth from external trauma as well as physical and chemical stimuli.^9^ Ameloblasts, as the key cells responsible for enamel development and mineralization, are lost upon tooth eruption.^9^ Consequently, enamel lacks any capacity for cellular repair, and once formed, it must function over a lifetime.^10^ However, due to local, systemic, genetic or environmental factors, abnormalities that originate during enamel formation are still prone to occur, which are referred to as developmental defects of enamel (DDE).^11–13^ DDE has a significant impact on patients since abnormal enamel can cause a series of problems, such as impaired masticatory function and aesthetic appearance, tooth loss and even maxillofacial development defects.^11,14^ Ameloblast differentiation is an essential process that guarantees normal enamel development and is regulated by various signalling pathways.^9^ Previous studies showed that circadian genes (*Bmal1*, *Clock*, *Per1*, *Per2*) and two markers of ameloblast differentiation, amelogenin (*Amelx)* and kallikrein-related peptidase 4 (*Klk4*), oscillated regularly in ameloblasts,^15^ which proved that clock gene products were detected in different stages during tooth germ development.^15,16^ Although circadian genes are expressed in ameloblasts and tooth germs, circadian rhythm, as an essential environmental regulating factor, its effects on enamel development need to be clarified. It is important to determine the correlation and regulatory mechanism between circadian rhythm and ameloblast differentiation as well as enamel formation.

Deletion of peroxisome proliferator-activated receptor gamma (*Pparγ*) in mice abolished or dampened circadian rhythm at both behavioural and cellular levels without affecting locomotor activity under regular LD conditions.^17^ The circadian gene *PER2* might regulate the timing and steps of cell lineage and cell fate.^18^ PER2 interacts with PPARγ and regulates the role of PPARγ in the transcriptional activity of target genes.^19^ Researchers have found that PPARγ agonists regulate AKT activity, while the effect of PPARγ on AKT is still controversial. Activation of PPARγ by agonists such as rosiglitazone and pioglitazone induced AKT phosphorylation, thus activating AKT.^20,21^ In contrast, another agonist, troglitazone, inhibited the phosphorylation of AKT.^22^ Moreover, AKT could phosphorylate β-catenin directly at Ser552, a site that differs from the regular phosphorylation target of GSK3β in the WNT upstream signalling pathway, and influence the subcellular localization of β-catenin, which is independent of WNT upstream signalling.^23–25^ Our previous study showed that translocation of β-catenin into the nucleus could inhibit mineralization in ameloblasts.^26^ Whether PER2, as an essential molecule of the circadian system, is involved in the ameloblast differentiation process via the above series of molecular signalling pathways needs to be clarified. Therefore, the present study focused on PER2-mediated regulation of ameloblast differentiation and enamel development.

## Results

### Circadian rhythm in ameloblast-lineage cells (ALCs) and similar fluctuating expression profiles of *Per2*, *Pparγ* and *Amelx* in mouse tooth germs

To investigate the circadian rhythm in ameloblasts and the correlation between *Per2* and *Pparγ*, the expression of *Per2*, *Pparγ* and *Amelx* was detected by qRT-PCR. *Per2* and *Bmal1* expression for 48 h was observed in a rhythmic profile of ALC cells after synchronization (Supplemental Fig. S1a). In addition, the mRNA expression of *Per2*, *Pparγ* and *Amelx* fluctuated in a similar pattern and exhibited higher expression at 20:45 than at 8:45 in mandibular first molar germs of postnatal day 2 (PN2) mice (Supplemental Fig. S1b).

### Circadian rhythm disruption in mice resulted in a reduction in enamel matrix formation and molecular dysregulation in ameloblasts

**Fig. 1:**
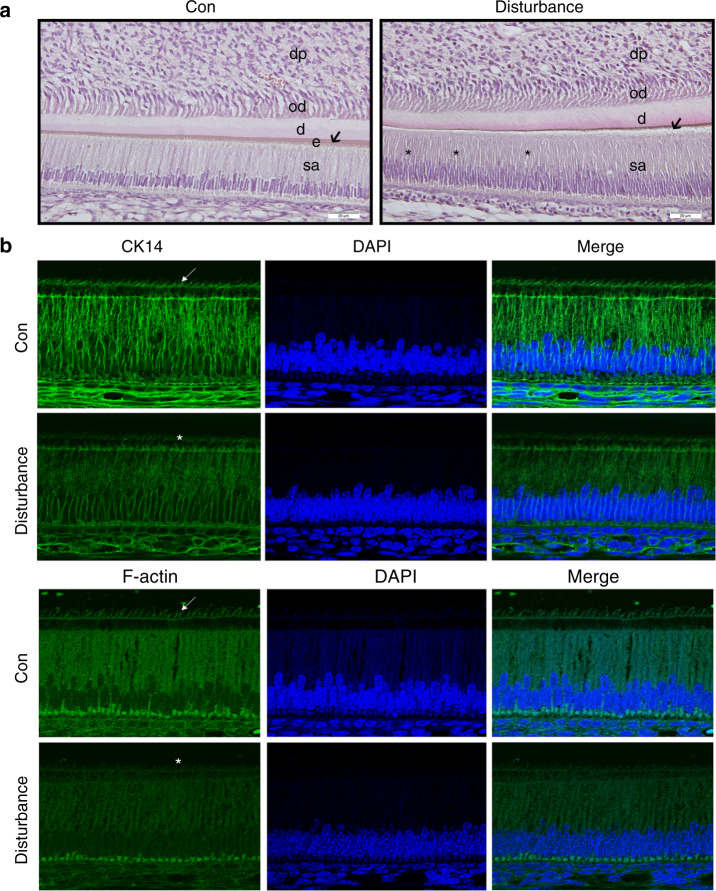
Decreased enamel matrix secretion and cytoskeletal molecules expression in the neonatal offspring of pregnant mice with circadian disruption. An environmental circadian disruption model was constructed in 10- to 12-week-old pregnant mice. **a** The mandibles of the offspring (PN5) were dissected. Compared to the control group, the enamel matrix secretion decreased (shown as black arrows), and there were some obvious voids (shown as black asterisks) among ameloblasts of mandibular incisor germs in the disturbance group. Bar = 20 μm; sa secretory ameloblast, e enamel, d dentin, od odontoblast, dp dental pulp. **b** Compared to the control group, the expression levels of CK14 and F-actin were reduced in ameloblasts, especially in Tomes’ processes in the disturbance group (shown as white arrows in the Con group and white asterisks in the disturbance group). Original magnification, ×100

**Fig. 2:**
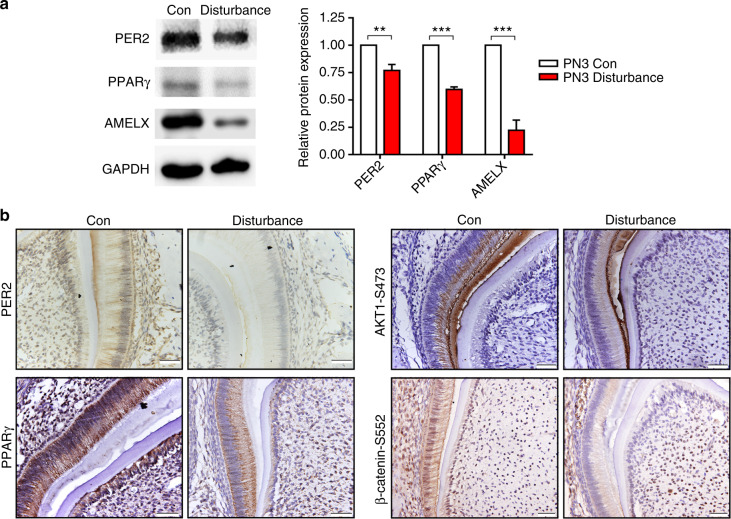
Dysregulated signalling molecules in the ameloblasts of neonatal offspring of circadian disruption pregnant mice. An environmental circadian disruption model was constructed in 10- to 12-week-old pregnant mice. **a** Total protein of mandibular first molar germs of the offspring (PN3) was extracted. Compared to the control group, the protein levels of PER2, PPARγ and AMELX decreased in the disturbance group. **b** The mandibles of the offspring (PN5) were dissected. Compared to the control group, the expression levels of PER2, PPARγ, AKT1-Ser473 and β-catenin-Ser552 were reduced in ameloblasts in the disturbance group. **P < 0.01; ***P < 0.001. Bar = 20 μm

To explore the underlying effect and molecular mechanism of circadian rhythm disruption involved in ameloblast differentiation in mice, 10- to 12-week-old pregnant mice were treated with or without exposure to environmental circadian disruption. All animal care and experimental protocols were ethically approved by the Institutional Animal Care and Use Committee of Wuhan University. After one week, the mandibles and tooth germs of neonatal mice were dissected and subjected to histology, immunofluorescence, western blot and immunohistochemistry. Compared to the control group, the circadian disturbance group showed less enamel matrix secretion (Fig. 1a), some obvious voids among ameloblasts (Fig. 1a) and decreased expression of cytokeratin 14 (CK14) and filamentous actin (F-actin) in ameloblasts, especially in Tomes’ process (Fig. 1b), suggesting the abnormal process of enamel matrix secretion and cell adhesion among ameloblasts. PPARγ was reported to participate in AKT phosphorylation and β-catenin regulation.^21,27,28^ Here, we found that the mandibular first molar germs of circadian disturbance mice exhibited reduced protein expression of BMAL1 (Supplemental Fig. S2a), PER2, PPARγ and AMELX (Fig. 2a) and decreased immunohistochemistry staining of BMAL1 (Supplemental Fig. S2b), PER2, PPARγ, AKT1-Ser473 and β-catenin-Ser552 in ameloblasts (Fig. 2b, Supplemental Fig. S2c). Reduced enamel matrix secretion and AMELX expression indicated that circadian disruption impeded the process of ameloblast differentiation in the offspring of pregnant mice, which might be associated with the PPARγ/AKT1/β-catenin signalling axis.

### PER2 activation by BMAL1 and potential regulatory signalling pathway prediction via KEGG enrichment analysis

**Fig. 3:**
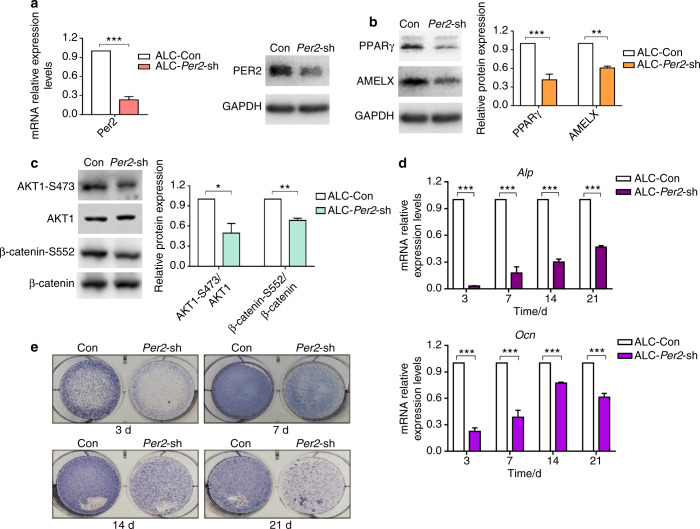
Altered signalling molecules expression and differentiation inhibition in *Per2*-knockdown ALC cells. **a** Construction of *Per2* knockdown ALC cell line. The knockdown efficiency of ALC-*Per2*-sh was examined by qRT-PCR and western blot. **b**, **c** Expression of PPARγ, AMELX, AKT1-Ser473 and β-catenin-Ser552 was reduced in ALC-*Per2*-sh. **d**, **e** ALC-Con cells and ALC-*Per2*-sh cells were cultured in differentiation-inducing medium. On days 3, 7, 14 and 21 of differentiation induction, in ALC-*Per2*-sh cells, ALP staining weakened (**e**), and the transcription levels of *Alp* and *Ocn* decreased (**d**). *P < 0.05; **P < 0.01; ***P < 0.001

*Per2-*knockdown ALC cells (ALC-*Per2*-sh), *Bmal1-*knockdown ALC cells (ALC-*Bmal1*-sh) and control cells (ALC-Con, transfected with empty vector) were successfully constructed and verified (Fig. 3a, Supplemental Fig. S3b). The expression of BMAL1 was slightly upregulated but showed no statistical significance in ALC-*Per2*-sh cells (Supplemental Fig. S3a), while ALC-*Bmal1*-sh cells showed decreased expression of PER2, AMELX and PPARγ compared with control cells (Supplemental Fig. S3b). Taken together, previous finding^5^ and our current study implied that BMAL1 led to PER2 activation and could regulate the expression of PPARγ mediated by PER2. Based on these findings, further study focused on PER2-mediated regulation of molecular changes and ameloblast differentiation.

RNA-seq was performed to compare the transcriptional profile of ALC-*Per2*-sh cells with that of ALC-Con cells. KEGG pathway analysis of differentially expressed genes (DEGs) identified the top 20 enriched pathways, which included the *Pi3k-Akt* signalling, *Wnt* signalling and *Pparγ* signalling pathways (Supplemental Fig. S3c). PPARγ, AKT1 and β-catenin were dysregulated in ameloblasts of tooth germs of neonatal mice with circadian disruption. Based on the abovementioned findings, the following exploration aimed to investigate PPARγ/AKT1/β-catenin, which might be involved in amelogenesis and correlate with each other and the circadian rhythm.

### *Per2* knockdown inhibited ALC cell differentiation with reduced expression of PPARγ, AKT1 and β-catenin phosphorylation and increased nuclear β-catenin

**Fig. 4:**
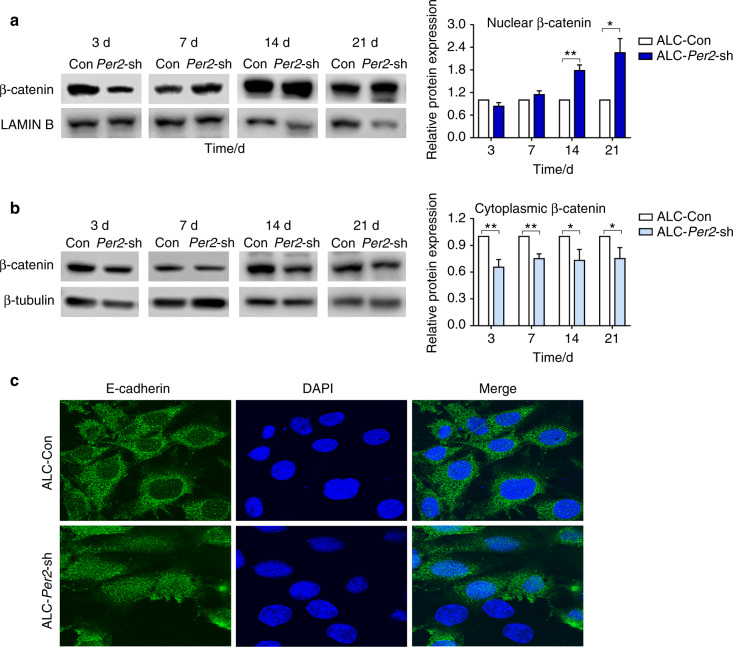
β-catenin translocated into the nucleus, and the subcellular localization of E-cadherin changed in *Per2*-knockdown ALC cells. **a**, **b** ALC-Con cells and ALC-*Per2*-sh cells were cultured in differentiation-inducing medium. On days 7, 14 and 21 of differentiation induction, in ALC-*Per2*-sh cells, β-catenin expression increased in the nucleus (**a**) and decreased in the cytoplasm (**b**) compared to that in control cells; **c** *Per2* knockdown altered the subcellular localization of E-cadherin in ALC cells. Original magnification, ×100. *P < 0.05; **P < 0.01

Consistent with the in vivo results, ALC-*Per2*-sh cells showed decreased protein expression of AMELX, PPARγ, AKT1-Ser473 and β-catenin-Ser552 compared with control cells (Fig. 3b, c). In the differentiation assay, ALC-*Per2*-sh cells showed inhibited differentiation activity with decreased mRNA expression of mineralization markers *Alp* and *Ocn* compared with ALC-Con cells (Fig. 3d). During the differentiation induction process, ALC-*Per2*-sh cells showed markedly weakened ALP staining compared to ALC-Con cells (Fig. 3e, Supplemental Fig. S4). Our previous study revealed that the activation of the WNT/β-catenin signalling pathway inhibited mineralization in ameloblasts.^26^ Here, we found that the expression of β-catenin was remarkably increased in the nucleus, especially at days 14 and 21 of differentiation induction (Fig. 4a), while it was decreased in the cytoplasm in ALC-*Per2*-sh cells (Fig. 4b). These results indicated that *Per2* depletion not only led to the decreased expression of AMELX, PPARγ, AKT1 and β-catenin phosphorylation but also caused the translocation of β-catenin into the nucleus and the subsequent repression of differentiation in ameloblasts.

### *Per2* knockdown altered the subcellular localization of E-cadherin in ALC cells

E-cadherin, a hallmark of cell adhesion, exhibited reorganized subcellular localization in ALC-*Per2*-sh cells, diffusing to the whole cell (Fig. 4c). This result implied the effect of *Per2* knockdown on the perturbation of ameloblast arrangement and secretion.

### PPARγ mediated the effect of PER2 on ALC cells through the AKT1/β-catenin signalling axis

**Fig. 5:**
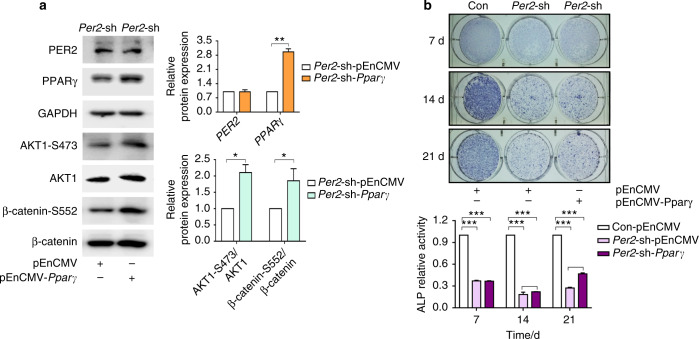
Overexpression of PPARγ partially rescued the altered signalling molecule expression and weakened ALP staining and ALP activity in *Per2*-knockdown ALC cells. **a** PPARγ was overexpressed in ALC-*Per2*-sh cells transfected with plasmids. With the increase in PPARγ expression, PER2 expression remained unchanged, while AKT1 and β-catenin phosphorylation levels were enhanced in the ALC-*Per2*-sh-pEnCMV-*Pparγ* group. **b** ALP staining and ALP activity were enhanced in ALC-*Per2*-sh-pEnCMV-*Pparγ* cells compared to those in ALC-*Per2*-sh-pEnCMV cells but still weakened compared to those in ALC-Con-pEnCMV cells. *P < 0.05; **P < 0.01; ***P < 0.001

**Fig. 6:**
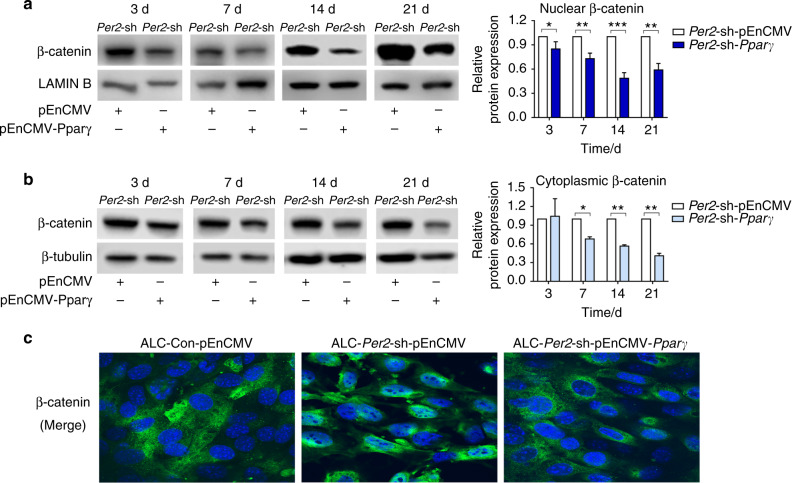
Overexpression of PPARγ reversed β-catenin subcellular localization in *Per2*-knockdown ALC cells. ALC-Con and ALC-*Per2*-sh were transfected with plasmids. **a**, **b** In the differentiation assay, the expression of β-catenin was obviously decreased in the nucleus (**a**) and cytoplasm (**b**) in ALC-*Per2*-sh-pEnCMV-*Pparγ* cells. **c** Cell immunofluorescence showed that β-catenin translocated into the nucleus in ALC-*Per2*-sh-pEnCMV cells compared with ALC-Con-pEnCMV cells but was reversed in ALC-*Per2*-sh-pEnCMV-*Pparγ* cells. *P < 0.05; **P < 0.01; ***P < 0.001

To further elucidate the regulatory relationship between PER2, PPARγ and AKT1 and β-catenin, *Pparγ* was overexpressed in ALC-*Per2*-sh cells transfected with plasmids. With the increase in PPARγ expression, PER2 expression remained unchanged, while AKT1 and β-catenin phosphorylation levels were enhanced in the ALC-*Per2*-sh-pEnCMV-*Pparγ* group (Fig. 5a). Combined with the above results, it was suggested that PPARγ was a downstream target of PER2 and positively regulated AKT1 and β-catenin phosphorylation. Moreover, the expression of β-catenin was significantly decreased in the nucleus in ALC-*Per2*-sh-pEnCMV-*Pparγ* cells compared to ALC-*Per2*-sh-pEnCMV cells (Fig. 6a, c**)** and presented immunofluorescence staining similar to that in ALC-Con-pEnCMV cells (Fig. 6c, Fig. S6). ALP staining and ALP activity were enhanced in ALC-*Per2*-sh-pEnCMV-*Pparγ* cells compared to those in ALC-*Per2*-sh-pEnCMV cells but still weakened compared to those in ALC-Con-pEnCMV cells, especially at days 14 and 21 of differentiation induction (Fig. 5b, Supplemental Fig. S5).

### PPARγ agonist rescued the impact of circadian disruption on incisor eruption in mice

**Fig. 7:**
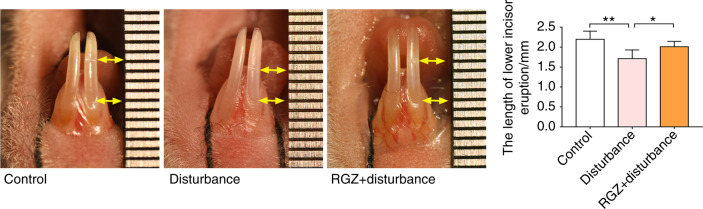
A PPARγ agonist reversed the decreased incisor eruption resulting from environmental circadian disruption in mice. Eight-week-old female mice were randomly divided into three groups: 12 h light/12 h dark + solvent (control); circadian disruption + solvent (disturbance); and circadian disruption + RGZ (RGZ + disturbance). DMSO (10%) in ddH_2_O served as a solvent. RGZ (PPARγ agonist rosiglitazone) was administered at 10 mg·kg^−1^ in solvent. Small dimples were created on the labial enamel surface of the alveolar bone crest, and the amount of lower incisor eruption was measured after 1 week. The length between the top and bottom yellow arrows indicates the amount of lower incisor eruption. The RGZ+ disturbance group showed comparable incisor eruption to that in the control group and significantly increased incisor eruption compared with that in the disturbance group (*n* = 6). *P < 0.05; **P < 0.01

To further elucidate the role of the circadian rhythm on incisor development and the rescue effect of PPARγ, a circadian disruption mouse model was established, and incisor eruption length was observed. Briefly, a dimple was created on the labial enamel surface close to the gingival margin in the mandibular incisors. The extent of incisor eruption was evaluated by measuring the length between the dimple and the labial gingival margin since mouse incisors continuously grow throughout life. Compared to the control group, the length of incisor eruption significantly decreased in the circadian disturbance group (Fig. 7, Supplemental Fig. S7) but was rescued by using a PPARγ agonist (Fig. 7).

Taken together, the in vitro and in vivo results indicated that PPARγ could partially rescue the inhibited differentiation activity associated with PER2 repression and β-catenin activation and reverse the effect of circadian disturbance on incisor development. These results suggested that PER2 regulated ameloblast differentiation through the PPARγ/AKT1/β-catenin signalling axis.

## Discussion

The circadian rhythm coordinates cellular and organismal metabolism, including regulation of heart rate, growth hormone secretion, bone modelling, lipid homeostasis and so on.^4,5^ Circadian gene products were detected during tooth development in mice, which indicated that circadian rhythm could play roles in odontogenesis.^16^ Nevertheless, the exact association between circadian rhythm and tooth development, especially ameloblast differentiation, needs to be clarified. In our study, we found that circadian disruption in mice resulted in molecular dysregulation, including decreased BMAL1 and PER2 and reduced enamel matrix formation during amelogenesis. The transcription of *Per2* was activated by BMAL1 according to a previous finding.^5^ In our study, *Bmal1*-knockdown ALC cells showed decreased protein expression of PER2 and PPARγ, while the expression of BMAL1 presented no significant variation in ALC-*Per2*-sh cells. Taken together, these results implied that BMAL1 led to PER2 activation and could regulate the expression of PPARγ mediated by PER2. Based on these findings, our study focused on PER2-mediated regulation of molecular changes and ameloblast differentiation. Through in vivo and in vitro experiments, we found inhibited ameloblast differentiation and decreased incisor eruption under circadian disturbance, which could be regulated by the PER2/PPARγ/AKT1/β-catenin signalling axis (Supplemental Fig. S8).

*Per2*, an important clock-controlled gene, is recognized as a key circadian regulator of development and diseases of many tissues.^29,30^ The expression of *Per2* and *Amelx* have been reported to oscillate in ameloblasts at regular circadian intervals (24 h),^15^ suggesting a correlation between circadian rhythm and ameloblast differentiation. In the present study, consistent with the above findings, we found that *Per2* mRNA followed circadian rhythms in ALC cells. Notably, *Per2*, *Pparγ* and *Amelx* mRNAs followed similar fluctuating expression patterns, which indicated that PPARγ might be involved in the process of ameloblast differentiation. In addition, previous studies demonstrated that PER2 is dampened when the circadian rhythm is disrupted.^31,32^ Here, in circadian disruption mice, we observed decreased expression of PER2, PPARγ, AKT1-Ser473, β-catenin-Ser552 and AMELX in ameloblasts, reduced enamel matrix secretion and decreased incisor eruption, showing the possible association between these molecules and circadian disturbed ameloblast differentiation.

PPARγ, a transcription factor of the ligand-activated nuclear receptor superfamily, plays an essential role in pulp cell survival, pulp homeostasis and dentin mineralization.^33,34^ Moreover, PPARγ is expressed in type II alveolar epithelial cells and is necessary for lung development,^35^ which indicates the involvement of PPARγ in epithelial cell differentiation. However, the role of PPARγ in ameloblasts, the most important epithelium-derived cell in enamel formation, remains unclear. Previous studies suggested that PER2 could directly regulate the transcriptional activity of PPARγ to target genes.^19^ Recent studies showed that AKT was related to ameloblast differentiation^36,37^ and could be regulated by PPARγ.^20–22^ In accordance with previous studies, we found that the knockdown of *Per2* reduced the expression of PPARγ and AKT1-S473 and that overexpression of PPARγ increased the phosphorylation of AKT1. In addition, in neonatal mice with circadian disruption, the expression of PER2, PPARγ and AKT1-Ser473 was decreased, accompanied by inhibited β-catenin-Ser552 expression. AKT was reported to phosphorylate β-catenin directly at Ser552, thus influencing its relocalization.^24,25^ Moreover, our previous studies have demonstrated that activated WNT/β-catenin signalling and accumulation of β-catenin in the nucleus could inhibit mineralization in ameloblasts and cementoblasts.^26,28^ In the present study, we found increased translocation of β-catenin into the nucleus and reduced differentiation activity in ALC-*Per2*-sh cells. Meanwhile, overexpression of PPARγ partially rescued the inhibited differentiation activity in ALC-*Per2*-sh cells, which was characterized by enhanced AKT1 and β-catenin phosphorylation levels, decreased expression of β-catenin in the nucleus and increased ALP staining and ALP activity in the ALC-*Per2*-sh-pEnCMV-*Pparγ* group. In addition, a PPARγ agonist reversed the inhibitory effect of circadian disturbance on incisor eruption. Based on these findings, it was suggested that a novel signalling network could regulate β-catenin subcellular localization and thus influence ameloblast differentiation through the PER2/PPARγ/AKT1/β-catenin signalling axis. In addition, as shown in our study, overexpression of PPARγ simply partially rescued the impeded ameloblast differentiation resulting from *Per2* knockdown, which implied that other signalling pathways were involved in the PER2-mediated ameloblast differentiation process. Therefore, further studies are required to determine the additional mechanism between PER2 and ameloblast differentiation.

In addition, β-catenin functions in cell adhesion associated with E-cadherin, which is relevant to cell polarity and migration, and functions as a trigger in regulating WNT/β-catenin signalling.^38,39^ In the present study, obvious voids and decreased expression of cytoskeletal proteins CK14 and F-actin in ameloblasts of neonatal mice with circadian disruption indicated a disturbance of the ameloblast arrangement, which might be explained by the dysregulation of β-catenin. The change in E-cadherin subcellular localization in *Per2*-knockdown ALC cells also implied an effect on the cell adherent junctions. Establishing and maintaining cell polarity is critical for cell migration, differentiation and transportation of the secretory matrix.^39^ On the one hand, β-catenin in the cytoplasm is involved in normal cell adherent junctions and polarity arrangements, which ensure the secretion and transport of ameloblasts.^9,38,40^ On the other hand, β-catenin translocating to the nucleus activates Wnt signalling, which functions to regulate enamel mineralization.

Overall, the study presented findings showing that circadian disruption in mice resulted in reduced enamel matrix formation and molecular dysregulation during amelogenesis. Moreover, it was further revealed that PER2, an essential molecule of the circadian system, exerted pivotal effects on the function and differentiation of ameloblasts, including amelogenin expression, cell junctions, enamel matrix secretion and mineralization, which were regulated by the PPARγ/AKT1/β-catenin signalling axis. However, the regulatory mechanism of circadian rhythm during tooth development has not been completely elucidated and needs further exploration.

In the limitations of the present study, the results highlighted that PER2 played an essential role in ameloblast differentiation through the PPARγ/AKT1/β-catenin signalling axis. The findings not only provide insights to understand the association and mechanisms between circadian rhythm and amelogenesis but also propose a new perspective for the prevention of developmental defects of enamel.

## Materials and Methods

### Reagents and antibodies

The reagents used for cell treatment were Lipofectamine 3000 (L3000015, Invitrogen), ascorbic acid (A4544, Sigma-Aldrich), sodium β-glycerophosphate (G9422, Sigma-Aldrich), and dexamethasone (D4902, Sigma-Aldrich). Primary antibodies included PER2 (NBP2-24616, Novus Biologicals), BMAL1 (NB100-2288, Novus Biologicals), PPARγ (A0270, ABclonal Technology, China), AKT1-Ser473 (AP0140, ABclonal Technology, China), β-catenin-Ser552 (AP0579, ABclonal Technology, China), AKT1 (A11016, ABclonal Technology, China), AMELX (ab153915, Abcam), β-catenin (A11343, ABclonal Technology, China), LAMIN B1 (A1910, ABclonal Technology, China), β-tubulin (PMK081M, BioPM, China), GAPDH (PMK042M, BioPM, China), CK14 (MA5-11599, ThermoFisher Scientific), F-actin (ab205, Abcam), and E-cadherin (sc-8426, Santa Cruz).

### Mouse models and groups

Kunming mice were purchased from the Provincial Centre of Disease Control (Wuhan, China) and housed at 22–24 °C and 55%–60% humidity under specific pathogen-free (SPF) conditions. Mice were maintained on a 12 h light/12 h dark cycle for at least 2 weeks before the study. Eight-week-old female mice were used in the experiments to estimate the incisor eruption rate: small dimples were created on the labial enamel surface of the alveolar bone crest, and the amount of lower incisor eruption was estimated after 1 week. The neonatal offspring of 10- to 12-week-old pregnant mice were used in the experiments to detect the development of tooth germs and ameloblasts. Mice had free access to a standard rodent chow diet and water and were randomly allocated into different groups. Normal conditions of a 12 h light/12 h dark cycle were disturbed by controlling lighting from 20:00 at night to 8:00 in the morning. The number and each group of mice (*n* = 6 per group) used in different experiments are specified in Supplemental Table 1. All experiments involving animals were performed according to the guidelines of the Institutional Animal Care and Use Committee of Wuhan University and approved by the Ethics Committee of the School and Hospital of Stomatology, Wuhan University (project identification code No. 2019A23).

### Tooth germ dissection and tissue preparation

The neonatal mice were decapitated using scissors. The lower jaw was separated from the rest of the head using scalpels under the microscope, and the lower jaws were kept in cold PBS on ice. The tooth germs of mandible first molars were isolated using microdissection forceps under the microscope. After dissection, total RNA or protein of the tooth germs was extracted for further experiments.

Mandibles were dissected from neonatal mice. The mandibles were divided in half along the midline and fixed in 4% paraformaldehyde (PFA) overnight, demineralized if necessary with 10% EDTA, dehydrated in a graded ethanol series, embedded in paraffin, and serially sectioned at 5 μm. The sections were stained with haematoxylin and eosin (HE) for histology and processed for immunohistochemistry and immunofluorescence using the antibodies described below.

### Cell culture, synchronization study, transfection and cell differentiation assay

The ameloblast-lineage cell (ALC) line^41^ (donation from Professor Souichi Koyota) was maintained in DMEM/F12 (HyClone) supplemented with 10% FBS (Gibco) and 1% penicillin-streptomycin (HyClone) for regular culture.

For the synchronization study, ALC cells were exposed for 2 h to serum-free medium to induce cell synchronization. After that, serum-free medium was changed to regular culture medium, and ZT0 (ZT: zeitgeber time, a standard time based on the period of an environmental synchronizer) was initiated after the medium change. The synchronized cells were cultured for 48 h and harvested every 6 h. Total RNA was extracted and analyzed by qRT-PCR.

For *Per2* knockdown, the lentivirus expression vectors *Per2*-sh and control (empty vector) were purchased from GeneChem (Shanghai, China). ALC cells were transfected with the lentivirus particles and named ALC-*Per2*-sh and ALC-Con. After 72 h of transfection, puromycin (Invitrogen) was used to select and maintain the stably transfected cell lines for further experiments. The protocol for *Bmal1* knockdown was the same as that for *Per2* knockdown.

For PPARγ overexpression, the plasmids pEnCMV-*Pparg*-m-3×FLAG and pEnCMV-MCS-3×FLAG (control vector) were purchased from Miaolingbio (Wuhan, China). ALC-Con and ALC-*Per2*-sh were transfected with plasmids diluted using Lipofectamine 3000 (Invitrogen) according to the manufacturer’s protocol.

When cells grew to near 60% confluence, regular culture medium was changed to differentiation medium containing 50 mg·L^−1^ ascorbic acid (Sigma-Aldrich), 10 mmol·L^−1^ sodium β-glycerophosphate (Sigma-Aldrich), and 10 nmol·L^−1^ dexamethasone (Sigma-Aldrich) for the differentiation assay. The cell samples were collected for qRT-PCR, western blot, ALP staining and ALP activity (Beyotime Biotechnology, China) at days 3, 7, 14, and 21.

For ALP staining, a BCIP/NBT Alkaline Phosphatase Colour Development Kit (Beyotime Biotechnology, China) was used according to the manufacturer’s protocols. Briefly, the cultured cells were fixed with 4% PFA for 15 min, rinsed with PBS, and incubated in BCIP/NBT staining solution for 15 min followed by rinsing with PBS again.

ALP activity was quantified using the Alkaline Phosphatase Assay Kit (Beyotime Biotechnology, China). The optical density was read spectrophotometrically at 405 nm. Relative ALP activity was normalized to the total protein concentration.

Each group of cells used in different experiments is specified in Supplemental Table 2.

### Immunohistochemistry and immunofluorescence

The sections mentioned above “tissue preparation” were used in this part. After deparaffinization and rehydration, the slices were treated by autoclaving in citric acid buffer (pH 6.0) for 5 min to expose antigens. Immunohistochemistry was performed with a streptavidin-peroxidase kit (ZSBO-Bio, Beijing, China). Samples were incubated overnight at 4 °C with the following primary antibodies: PER2 (1:200), BMAL1 (1:500), PPARγ (1:400), AKT1-Ser473 (1:300), and β-catenin-Ser552 (1:400). After incubation, the samples were managed according to the manufacturer’s protocols. The colour reaction was performed using diaminobenzidine (DAB, Maxim, Fujian, China). The sections were then counterstained with haematoxylin.

The immunohistochemistry paraffin sections were subjected to microscopic analysis. Light yellow to brown staining was recorded as positive immunostaining. The mean optical density of proteins was measured by Image-Pro Plus 6.0 (Media Cybernetics, USA).^42^

For CK14 and F-actin immunofluorescence staining, paraffin sections were treated with anti-CK14 mouse monoclonal antibody (1:500) followed by goat anti-mouse IgG secondary antibody Alexa Fluor® 488 (1:200, ZF-0512, ZSBO-Bio, China) and anti-F-actin mouse monoclonal antibody (1:50) followed by FITC-conjugated goat anti-mouse IgM (1:200, 33221ES60, YEASEN, Shanghai, China).

For ALC cell immunofluorescence, cells were fixed with 4% PFA for 20 min, rinsed with PBS, and blocked in serum in a 37 °C incubator for 1 h. Anti-E-cadherin mouse monoclonal antibody (1:50) and anti-β-catenin (1:100) rabbit antibody were used overnight incubation at 4 °C. Cells were then incubated with goat anti-mouse and goat anti-rabbit IgG secondary antibody Alexa Fluor® 488 (1:200, ZF-0512, ZF-0511, ZSBO-Bio, China) for 1 h at room temperature followed by staining with DAPI.

The immunofluorescence paraffin sections and cells were analyzed with a confocal laser scanning microscope (Olympus FV1200, Japan).

### Western blot

Total proteins of tooth germs and ALC cells were extracted using RIPA buffer supplemented with 1 mM PMSF. NE-PER Nuclear and Cytoplasmic Extraction Reagents (Thermo Fisher Scientific) were used to extract cytoplasmic and nuclear proteins according to the manufacturer’s protocol. The concentration of all protein samples was measured by a BCA protein assay kit (Beyotime Biotechnology, Shanghai, China). Equal quantities of proteins were subjected to SDS-PAGE and then electrophoretically transferred onto PVDF membranes. Specific primary antibodies included PER2 (1:1 000), BMAL1 (1:1 000), PPARγ (1:1 000), AKT1-Ser473 (1:500), AKT1 (1:500), AMELX (1:1 000), β-catenin (1:1 000), β-catenin-Ser552 (1:1 000), LAMIN B1 (1:500), β-tubulin (1:1 000) and GAPDH (1:5 000). The immunoreactive proteins were detected by an ECL system (Thermo Fisher Scientific). GAPDH was used as the normalized control for total protein lysis buffer. LAMIN B1 was set as the normalized control for nuclear protein lysis buffer, while β-tubulin was set as the normalized control for cytoplasmic protein lysis buffer.

### RNA-seq and quantitative real-time PCR (qRT-PCR)

Total RNA of ALC-Con and ALC-*Per2*-sh cells was extracted using TRIzol reagent (YEASEN, Shanghai, China) and then used for mRNA sequencing (BGI-Shenzhen, China). The criterion for differential gene expression was set as |fold change| > 1 and false discovery rate less than 0.05.

Total RNA of tooth germs and ALC cells was extracted using TRIzol reagent. One microgram of total RNA was reverse-transcribed into cDNA using Hieff® 1st Strand cDNA Synthesis SuperMix for qPCR (YEASEN, Shanghai, China). qRT-PCR was performed using Hieff® qPCR SYBR Green Master Mix (YEASEN, Shanghai, China). The relative gene expression was calculated using the equation 2^−Δ(ΔCt)^, where ΔCt = Ct (mRNA) − Ct (β-actin). PCR primers for each gene are listed in Supplemental Table 3.

### Statistical analysis

All quantitative experiments were performed in triplicate. GraphPad Prism 7.0 software was used for data analyses and graphing. All quantitative data are presented as the mean ± s.e.m. (standard error of mean). Statistical comparisons between two experimental groups were analyzed by unpaired, two-tailed Student’s *t*-test. Multiple comparison tests were performed using one-way ANOVA with Bonferroni’s test for more than two groups. For all tests, a *P* value < 0.05 was considered statistically significant.

## Supplementary information

supplemental materials

## Data Availability

The data used and/or analysed during the current study are contained within the manuscript or available from the corresponding author on reasonable request.
