# Supplementary material for: PER2-mediated ameloblast differentiation via PPARγ/AKT1/β-catenin axis
Source: Int J Oral Sci. 2021 May 19;13:16. doi: 10.1038/s41368-021-00123-7 (PMC8134554; doi:10.1038/s41368-021-00123-7)
Supplement: Supplementary file 1 — supplemental materials [file 41368_2021_123_MOESM1_ESM.docx]

**PER2-mediated ameloblast differentiation via PPARγ/AKT1/β-catenin axis**

Wushuang Huang, Xueqing Zheng, Mei Yang, Ruiqi Li, Yaling Song

**Table S1. The mice in different groups**

| **Mice** | **Groups** | **Experiments** |
| --- | --- | --- |
| neonatal offspring of 10- to 12-week old pregnant mice | Raised in normal condition (12h light/ 12h dark). | Extracted tooth germ RNA used for testing the transcription of *Per2*, *Pparγ*, *Amelx* by qRT-PCR. |
| neonatal offspring of 10- to 12-week old pregnant mice | Con (n=6): mice raised in normal condition;  Disturbance (n=6): mice raised in circadian rhythm disruption condition. | Extracted tooth germ proteins were used for western-blot and mandibles for histology, IHC and IF. |

**Table S2. Cells cultured in different groups**

| **Cells Goups** | **Culture condition** | **Experiments** |
| --- | --- | --- |
| ALC cells | After inducing cell synchronization, serum-free medium was changed to regular culture medium for 48 h cell culture and cells were harvested every 6 h. | *Per2* and *Bmal1* rhythmic expression was detected by qRT-PCR. |
| ALC- Con;  ALC-*Per2*-sh | Cells cultured in regular medium for 3 days and the medium was changed every 2 day. | Extracted RNA and proteins of cells were used for qRT-PCR and western-blot, and fixed cells for IF. |
| ALC- Con;  ALC-*Per2*-sh | Cells cultured in differentiation inducing medium containing 50 mg L^-1^ ascorbic acid, 10 mM sodium β-glycerophosphate, and 10 nM dexamethasone for mineralization assay. Cells cultured for 3, 7, 14, 21 days and the medium was changed every 2 day. | RNA and cytoplasmic and nuclear protein were extracted for qRT-PCR and western-blot, and fixed cells for ALP staining. |
| ALC-*Per2*-sh-pEnCMV;  ALC-*Per2*-sh-pEnCMV-*Pparγ* | Cells cultured for 3 days and the medium was changed every 2 day. | Protein of different cell groups were extracted for western-blot. |
| ALC-Con-pEnCMV;  ALC-*Per2*-sh-pEnCMV;  ALC-*Per2*-sh-pEnCMV- *Pparγ* | Cells cultured for 3 days and the medium was changed every 2 day. | cells immunofluorescence |
| ALC-*Per2*-sh-pEnCMV;  ALC-*Per2*-sh-pEnCMV- *Pparγ* | Cells cultured in differentiation inducing medium; Cells cultured for 3, 7, 14, 21 days, and the medium was changed every 2 day. | Cytoplasmic and nuclear protein were extracted for western-blot. |
| ALC-Con-pEnCMV;  ALC-*Per2*-sh-pEnCMV;  ALC-*Per2*-sh-pEnCMV- *Pparγ* | Cells cultured in differentiation inducing medium; Cells cultured for 7, 14, 21 days, and the medium was changed every 2 day. | Fixed cells for ALP staining and cells lysate used for ALP activity. |
| ALC- Con;  ALC-*Bmal1*-sh | Cells cultured in regular medium for 3 days and the medium was changed every 2 day. | Extracted RNA and proteins of cells were used for qRT-PCR and western-blot. |

**Table S3. Primers used in qRT-PCR**

| **Gene** | **Forward primers (5’-3’)** | **Reverse primers (5’-3’)** |
| --- | --- | --- |
| *β-actin* | AACCCTAAGGCCAACCGTGAAA | CGTACATGGCTGGGGTGTTGA |
| *Per2* | CTTGATGCTCGCCATCCACA | TATCTTCCTGCTCCACGGGT |
| *Pparγ* | ATTGAGTGCCGAGTCTGTGG | CCCTTGCATCCTTCACAAGC |
| *Amelx* | CCTGGATTTTGTTTGCCTGCCTC | ATAACCAGGGCTTCCAGGATGAG |
| *Alp* | AGTAACCGCTGCCCGAATC | GGACCTGAGCGTTGGTGTTAT |
| *Ocn* | TGACCTCACAGATGCCAAGC | GCCGGAGTCTGTTCACTACC |
| *Bmal1* | AACGGGGAAATACGGGTGAA | GCGAGGTGTCCTATGTCGTC |

**
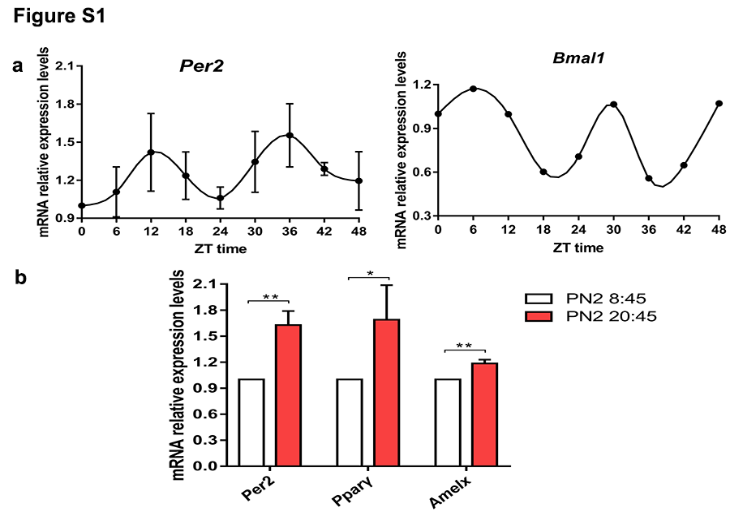
**

**Figure S1.** **Circadian rhythm in ALC cells, and similar fluctuated expression profile of *Per2*, *Pparγ* and *Amelx* in mice tooth germs**

(a) The synchronized ALC cells were cultured for 48 h and harvested every 6 h. *Per2* and *Bmal1* mRNA exhibited rhythmic expression pattern in ALC cells; (b) The mRNA transcription levels of *Per2*, *Pparγ* and *Amelx* were higher at 20:45 than that at 8:45 in mandibular first molar germs of postnatal day 2 (PN2) mice. * *P* < 0.05; ** *P* < 0.01

**
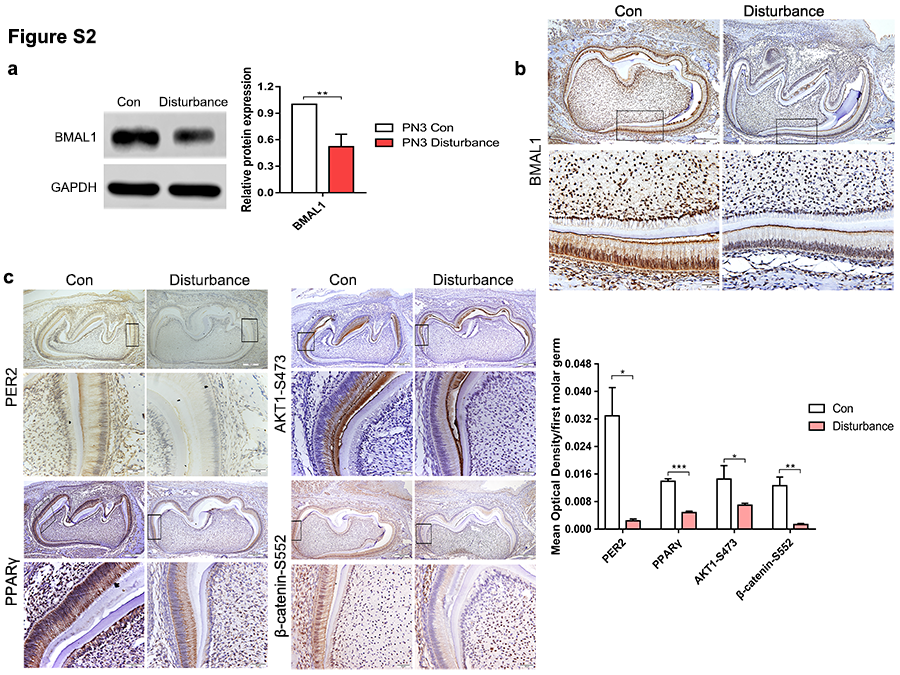
**

**Figure S2. Decreased molecules expression in molar germs and ameloblasts of environmental circadian disrupted mice**

An environmental circadian disruption model was constructed in 10-12-week old pregnant mice. Total protein of mandibular first molar germs of the offspring (PN3) were extracted and mandibles of the offspring (PN5) were dissected. (a, b) Compared to the control group, the protein level of BMAL1 decreased and the immunostaining of BMAL1 in ameloblasts was obviously weaker in the disturbance group; (c) The immunostaining of PER2, PPARγ, AKT1-Ser473 and β-catenin-Ser552 in ameloblasts were semi-quantified by Image-Pro Plus 6.0, the expression levels of positive cells were significantly reduced in the disturbance group. * *P* < 0.05; ** *P* < 0.01; *** *P* < 0.001. Bar = 100 μm / 20 μm.

**
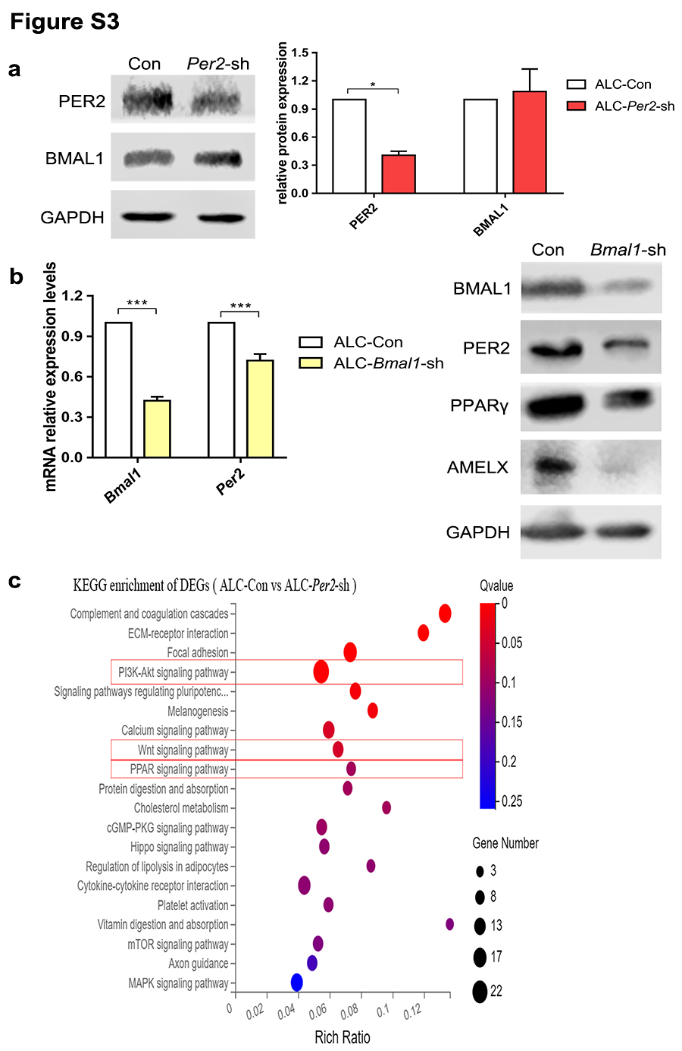
**

**Figure S3.** **BMAL1 acts upstream of PER2 in ALC cells and potential regulating signaling pathways prediction via KEGG enrichment analysis**

(a) The expression of BMAL1 was slightly up-regulated but with no statistical significance in ALC-*Per2*-sh cells; (b) The knockdown efficiency of ALC-*Bmal1*-sh was examined by qRT-PCR and western blot. Expression of PER2, PPARγ and AMELX were reduced in ALC-*Bmal1*-sh; (c) KEGG pathway analysis of differentially expressed genes (DEGs) identified the top 20 enriched pathways.

* *P* < 0.05; *** *P* < 0.001.

**
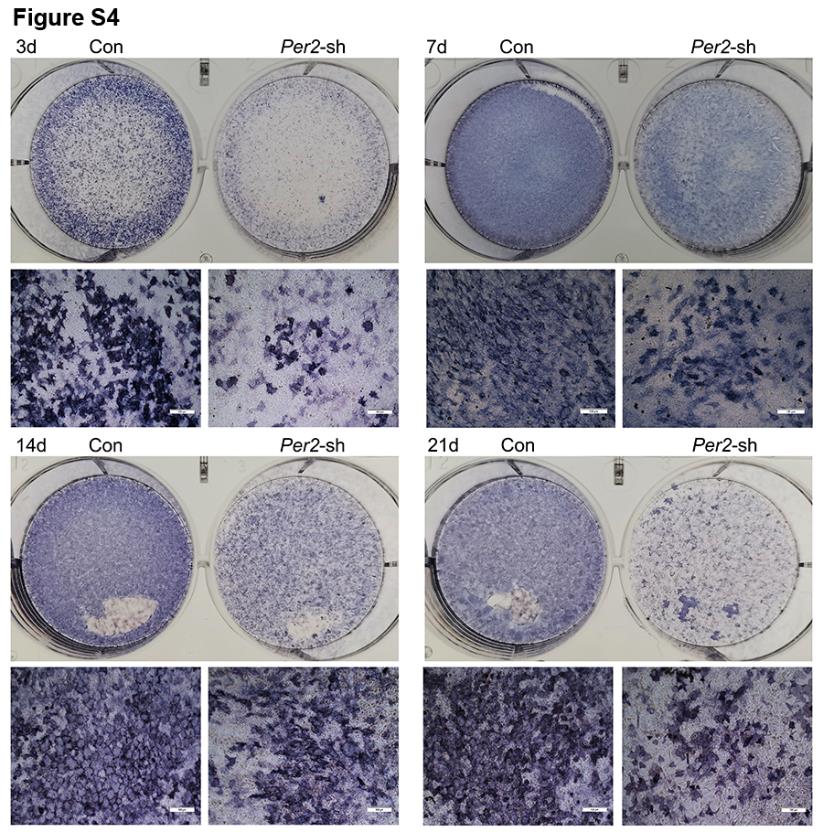
**

**Figure S4. ALP staining was obviously weaker in *Per2*-knockdown ALC cells than in ALC-Con cells**

ALC-Con cells and ALC-*Per2*-sh cells were cultured in differentiation inducing medium. On day 3, 7, 14 and 21 of mineralization induction, ALP staining weakened in ALC-*Per2*-sh cells. Bar = 100 μm

**
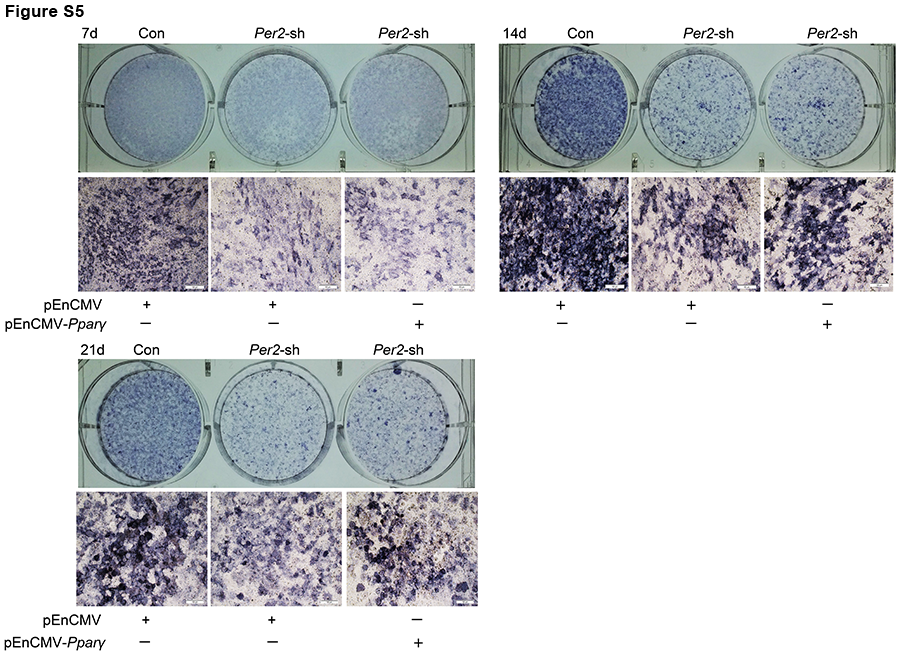
**

**Figure S5. Overexpression of PPARγ partially rescued the weakened ALP staining in *Per2*-knockdown ALC cells**

ALC-Con and ALC-*Per2*-sh were transfected with plasmids. ALP staining slightly enhanced in ALC-*Per2*-sh-*Pparγ* compared to those in ALC-*Per2*-sh-Con but still weakened compared to those in ALC-Con cells especially for D14 and D21 cells. Bar = 100 μm

**
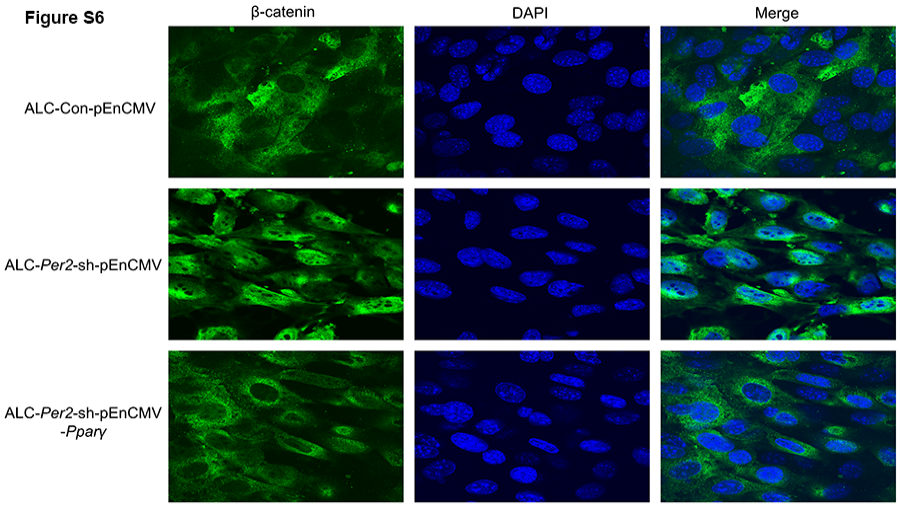
**

**Figure S6. Overexpression of PPARγ reversed β-catenin subcellular location in *Per2*-knockdown ALC cells**

Cell immunofluorescence showed that β-catenin translocated into the nucleus in ALC-*Per2*-sh-pEnCMV cells compared with those in ALC-Con-pEnCMV but was reversed in ALC-*Per2*-sh-pEnCMV-*Pparγ* cells. Original magnification, × 100.


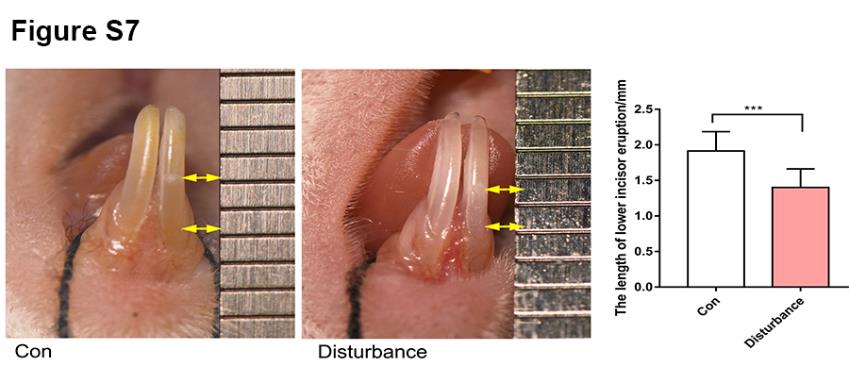


**Figure S7. Decrease incisor eruption in circadian disruption mice**

Small dimples were created on the labial enamel surface of alveolar bone crest, and the amount of lower incisor eruption was measured after 1 week. The length between top and bottom yellow arrows indicated the amount of lower incisor eruption. Compared to the control group mice, the amount of incisor eruption was statistically reduced in circadian disruption group mice (n=6).

*** *P* < 0.001.


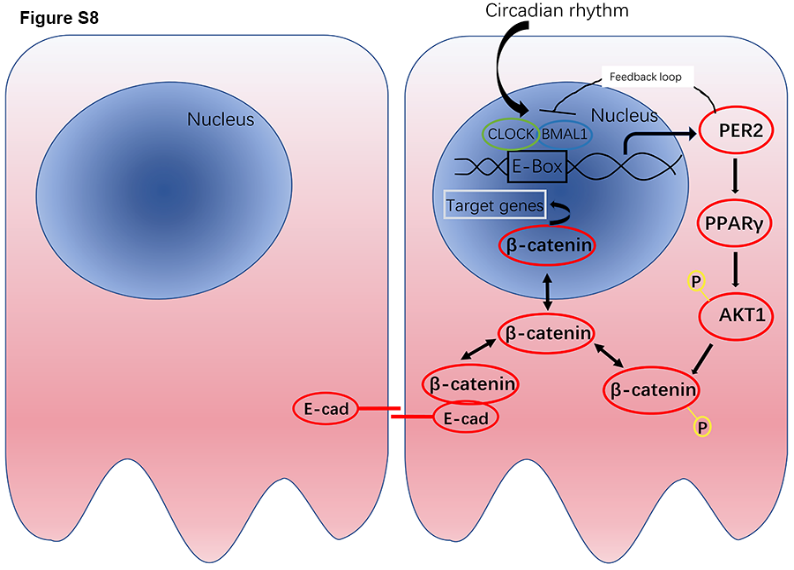


**Figure S8. Schematic diagram of PER2 regulating ameloblast differentiation mediated by PPARγ/AKT1/β-catenin signaling axis.**

In our study, when circadian rhythm was disrupted, the expression of PER2 was dampened, resulting in the down-regulated expression of PPARγ and reduced AKT1 and β-catenin phosphorylation. Cytoplasmic β-catenin functions different ways: it could be involved in adherent junctions associated with E-cadherin, and could be phosphorylated in the cytoplasm or translocated into the nucleus to trigger target genes expression. *Per2* depletion not only led to decreased expression of PPARγ, AKT1 and β-catenin phosphorylation but also caused the translocation of β-catenin into the nucleus and the subsequent differentiation repression in ameloblasts; and overexpression of PPARγ could partially rescue these molecular changes resulting from *Per2*-knockdown in ALC cells.

**
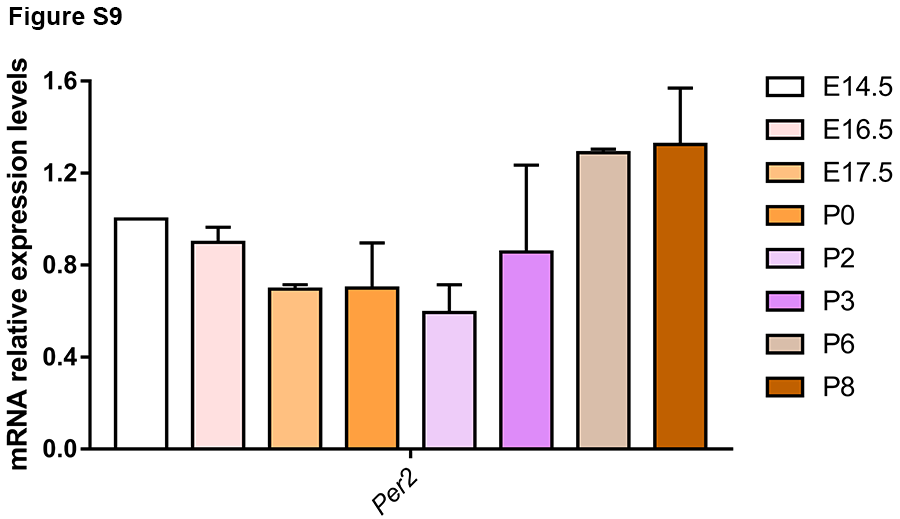
**

**Figure S9. The mRNA expression level of *Per2* in the mice mandibular first molar germs at different time points**

**
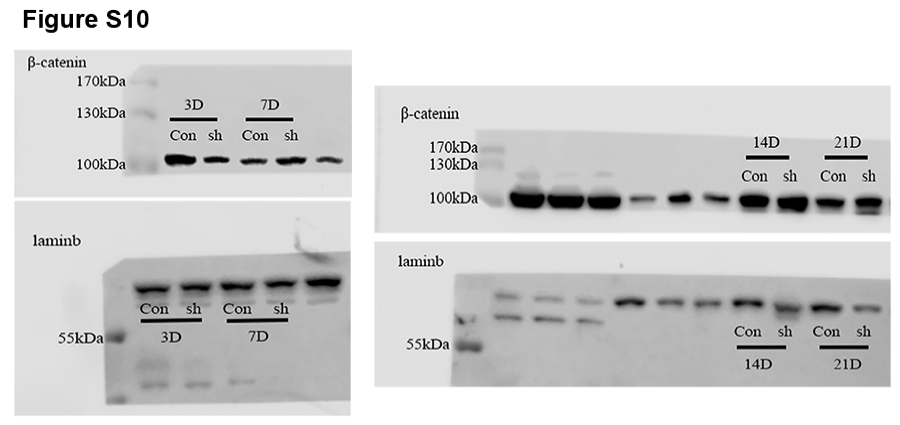
**

**Figure S10. The original western blot figure of Figure 4a**
